# Punishment as Moral Fortification and Non-Consensual Neurointerventions

**DOI:** 10.1007/s10982-018-09341-3

**Published:** 2019-02-08

**Authors:** Areti Theofilopoulou

**Affiliations:** Oxford Uehiro Centre for Practical Ethics, Faculty of Philosophy, University of Oxford, Suite 8, Littlegate House, 16-17 St Ebbes Street, Oxford, OX1 1PT, UK

## Abstract

The purpose of this paper is twofold. First, I defend and expand the Fortificationist Theory of Punishment (FTP). Second, I argue that this theory implies that non-consensual neurointerventions – interventions that act directly on one’s brain – are permissible. According to the FTP, punishment is justified as a way of ensuring that citizens who infringe their duty to demonstrate the reliability of their moral powers will thereafter be able to comply with it. I claim that the FTP ought to be expanded to include citizens’ interest in developing their moral powers. Thus, states must ensure that their citizens develop their moral reliability, not only because they must enforce their citizens’ compliance with certain duties, but also because states have the duty to maintain the conditions for stability and satisfy their citizens’ interest in developing their moral powers. According to this account of the FTP, if neurointerventions are the only or best way of ensuring that offenders can discharge their fortificational duties, states have strong reasons to provide these interventions.

Certain criminal offenders are required or given the option to undergo neurointerventions – interventions that act directly on one’s brain – either as part of their sentence or as a condition of their parole. Most commonly, this occurs in the form of lowering sex offenders’ testosterone through the administration of hormonal agents, such as triptorelin.^1^ Due to the increasing possibilities of using such interventions, there is a growing academic debate on the moral questions regarding their potential uses to achieve greater empathy and impulse control in offenders.

The purpose of this paper is twofold. First, I defend and expand the Fortificationist Theory of Punishment. Second, I argue that, according to the Fortificationist Theory, non-consensual neurointerventions are permissible. In developing this argument, I first present Jeffrey Howard’s case for the Fortificationist Theory of Punishment.^2^ According to this view, criminal offenders who violate just laws demonstrate that they cannot – or are not willing to – act on the basis of their moral powers. When citizens fail to discharge their duty to demonstrate the reliability of their moral powers, states ought to ensure that they will comply with it in the future. Apart from citizens’ fortificational duties, I argue that there are two further considerations that collectively serve to justify punishment according to the Fortificationist Theory: these are a state’s duty to promote the collective good of stability and the interest that all individuals have in developing their moral powers. Thus, in this interpretation of the rehabilitative approach to punishment, the purpose of a criminal justice system must be to foster offenders’ capacity to both understand and be motivated by the requirements of justice.

An interesting and so far unexplored implication of the Fortificationist Theory is that non-consensual neurointerventions are morally permissible and perhaps even morally required, under certain conditions. I develop this argument in Sect. II, where I argue that there are at least three strong reasons in favour of neurointerventions once we accept the Fortificationist Theory.

In the third and final section of the paper I examine three objections to my argument. The first objection raises the possibility that non-consensual neurointerventions are impermissible because they necessarily violate offenders’ basic liberties. If successful, this objection would imply that, even if we accept that the Fortificationist Theory of Punishment produces certain reasons in favour of neurointerventions, these reasons are decisively outweighed by the reasons we have to protect individuals’ mental freedom and bodily rights. According to the second objection, neurointerventions can never be proportionate to the aims that justify punishment according to the Fortificationist Theory. This is because of the potential harmful side-effects of these interventions, because they infringe individuals’ autonomy, and because they might cause a disruption in a person’s psychological continuity across time. The third and final objection, which is implicitly raised by Howard, draws on an analogy between indoctrination and neurointerventions and states that, if indoctrination is impermissible and inconsistent with the proper reading of the Fortificationist Theory of Punishment, then so are neurointerventions. By responding to these objections, I conclude that the three reasons in favour of neurointerventions retain their force. Although my argument is by no means sufficient to establish the all-things-considered permissibility of neurointerventions, mainly due to the non-ideal conditions that characterise our world here and now, this discussion shows that this argument must be taken seriously by proponents of the Fortificationist Theory of Punishment.

## I The Fortificationist Theory of Punishment

According to Howard, the Fortificationist Theory of Punishment offers a new interpretation of rehabilitative approaches to criminal justice, which is immune to the standard objection raised against such approaches – that they fail to treat offenders as moral agents rather than blameless victims. This interpretation states that, together with our duty to refrain from violating others’ rights, we – as moral agents – also have a duty of reliability – that is, a duty to ‘maintain the dependability of our moral capacities’.^3^ This is because, unless we maintain this dependability, we risk succumbing to desires that may lead us to violate others’ rights. In this way, the duty of reliability is inextricably linked with our duty to refrain from violating others’ rights. In Howard’s words,

Doing one’s duty is often not experienced as a matter of flicking a switch. There are potential psychological hurdles that agents may well encounter along the way. And we typically judge that it is the agent herself who is primarily responsible for overcoming them. Thus our primary moral duties to perform or refrain from performing certain acts are, in fact, often accompanied by ‘waves’ of fortificational duties whose fulfilment positions the agent to live up to the associated primary duties successfully.^4^

Such fortificational duties may include pursuing counselling or changing one’s lifestyle if that is necessary to ensure one’s compliance with one’s primary moral duties.

Thus, when moral agents violate just laws, they reveal that they have violated two related duties. First, they have clearly violated their duty to refrain from acting unjustly. Second, they show that they have failed to discharge their duty to remain trustworthy by maintaining the dependability of or by *fortifying* their moral capacities. The violation of these connected duties produces a distinctive answer to the question of the justifying aim of punishment. That is, states may intervene through punishment in order to enforce the duty that ‘offenders have to reduce their own likelihood of recidivism’.^5^ Given that the criminal offenders in question have failed to comply with their own fortificational duties, states ought to take on the task of fortifying those offenders’ moral powers, to ensure that they become capable of maintaining their dependability.

Within the Rawlsian context in which Howard sets his argument, the two moral powers that all moral agents have a duty to fortify are what Rawls calls ‘reasonableness’ – the capacity for a sense of justice – and ‘rationality’ – the capacity to form, revise, and pursue a conception of the good. The moral power that is relevant in the context of the criminal law is reasonableness – the capacity for a sense of justice, which includes the *epistemic* aspect of understanding the requirements of justice and the *motivational* aspect of being willing to comply with those requirements.^6^ The Fortificationist Theory of Punishment thus implies that, when faced with a criminal offender who has failed to fortify her moral powers, states must enforce that offender’s duty by targeting the epistemic or the motivational aspect of her reasonableness, or both. This must be done in the appropriate way, given that, for example, education may be the appropriate way to fortify one’s lack of moral understanding, but cases of motivational deficiencies may require different interventions, such as counselling.

Howard’s formulation of the Fortificationist Theory of Punishment effectively rests on the premise that states have a responsibility to ensure that all citizens comply with their moral duties. I argue that there are three distinct reasons for endorsing this claim.

Clearly, the claim that states can intervene when individuals fail to comply with their moral duties is too broad, for, in some cases, such intervention may not be justified. Enforcing honesty in individuals’ private and consensual relationships would be one of these cases. Thus, we ought to understand the fortificationist position to be claiming that the duties that are transferred to the state when individuals fail to comply with them are those that exist to protect the rights and liberties that ought to be legally protected. It is not possible here to develop an account of which rights and liberties ought to be legally protected, but it suffices to note that *if* an individual’s right or liberty ought to be legally protected, then states have a duty to intervene when the law protecting that right or liberty is violated. According to this interpretation, the first reason why states have a responsibility to enforce compliance with moral duties, such as the fortificational duties that are linked to primary duties, is that states themselves have their own duty to protect *individuals*’ rights and liberties.

There exists, however, a second reason that supports the view that states bear this responsibility. This reason springs from states’ duty to promote the *collective* good of maintaining a well-ordered society. For, if citizens have good reasons to doubt their co-citizens’ commitment to justice, the grounds for stable and fair social cooperation become shaky. Although the appeal to stability is connected to the appeal to individuals’ rights and liberties (because one reason why we desire stability is that it ensures the protection of our rights), the two claims are distinct: the former is a societal good, whereas the latter is an individual good. This distinction then shows that states’ responsibility to enforce individuals’ compliance with their fortificational duties has a second source, which is states’ duty to promote the stability of their society.

Finally, I suggest that a third reason favouring the Fortificationist Theory is the interest that all persons have in developing their moral powers, and the correlative duty that states have to provide the conditions that enable their citizens to satisfy this interest.

The claim that we have an interest in developing our moral powers is implicitly endorsed by most moral theories that attribute special value to moral agency. Although some philosophers might cast this claim in perfectionist terms, which may open up worries about paternalism, this need not be the case, as we can accept it without defending a specific conception of the good, such as a specific view of human flourishing.^7^ This is because our interest in developing our moral powers is grounded in our interest in participating in social cooperation. Given that cooperating requires a certain kind of reciprocity, those who fail to engage in such relationships by virtue of their failure to fortify their moral powers lack the capacity to participate in social cooperation. This implies that part of the justification that an anti-perfectionist state can give citizens when enforcing their duties is that they have an interest in being moral agents who can participate in reciprocal relationships. This justification can be accepted without leading us to the undesirable implication that a state can enforce anything that is in its citizens’ interests construed in perfectionist terms. Thus, a third reason why states have a duty to enforce the fortificational duties of citizens when the citizens fail to enforce the duties themselves is that citizens have an interest in possessing the moral powers that enable them to participate in social cooperation.

Of course, this does not imply that states ought to constantly fortify their citizens’ moral powers. Rather, moral powers must be understood as ‘range properties’ that are possessed by all those who have them beyond some minimum threshold. A person’s interest in developing her moral powers therefore calls for fortification only when there is clear evidence that her moral powers have not reached the relevant threshold. It follows that states have an interest-based duty to fortify a citizen’s moral powers only when this is the case. Indeed, this seems to be one justification for the duty that states have to provide good, mandatory education for children and appropriate pedagogical policies for individuals with cognitive disabilities.^8^

## II Fortificationism and Neurointerventions

Recall that, according to my expanded version of the Fortificationist Theory of Punishment, there are three distinct reasons that justify state intervention when citizens fail to comply with their fortificational duties. The first reason is that states have a duty toward *individuals* to ensure the protection of their basic rights and liberties; when a citizen fails to comply with her enforceable duties to her co-citizens, states ought to step in and enforce compliance. The second reason is that states have a duty to ensure that there are the appropriate social conditions that make stable and fair cooperation possible. Clearly, the prospect of such cooperation is undermined when citizens have good reason not to trust each other. The third and final reason that supports state intervention when citizens fail to fortify their moral powers is that states have a duty to satisfy their citizens’ interest in possessing the moral powers to the degree that enables them to participate in social cooperation.

As Howard has claimed, it seems straightforward that since punishment is justified by appeal to the aim of fortifying criminal offenders’ moral powers, the modes of punishment that states ought to use must be expected to have the required fortificational effects. As a result, instead of permitting incarceration as we observe it in current societies, states should turn to policies such as cognitive behavioural therapy assignments, discussion seminars, and prisons that are ‘replicas of the outside world … providing offenders with substantive access to work, education, and treatment for substance abuse’.^9^

I suggest, however, that a more controversial and so far unexplored implication of the Fortificationist Theory is that non-consensual neurointerventions are also a justifiable mode of punishment. As with therapy and other modes of punishment, the justifiability of neurointerventions would be subject to the condition that they are expected to have the desired effect of fortifying the offender’s moral powers to the requisite degree.

The most widely discussed case of neurointerventions in the context of criminal justice is that of anti-libidinal pharmacological agents administered to sex offenders. There is wide evidence that agents such as cyproterone acetate (CPA), medroxyprogesterone acetate (MPA), and gonadotrophin-releasing hormone (GnRH) agonists can reduce one’s testosterone levels, thereby inducing chemical castration.^10^ Similarly, it has been observed that a side-effect of selective serotonin reuptake inhibitors (SSRIs), which are typically used to treat depression and anxiety, is a decrease in patients’ libido, although there is no consensus on the explanation of this side-effect.^11^ Although there is still disagreement on the effectiveness of these agents, there are studies that indicate that they significantly decrease the probability of reoffending.^12^ These effects are reversible, as maintaining them requires constant treatment.^13^

As a second example, consider pharmacological agents that target serotonin levels. Lower levels of serotonin have been associated with aggression and antisocial behaviour; for instance, many individuals with aggressive and antisocial behaviour have mutations in the gene coding for the 5-HT2B serotonin receptor, or variations in the gene coding for MAOA, a brain enzyme that is crucial for breaking down serotonin.^14^ Although the causal relationship between serotonin and aggressive or antisocial behaviour is not completely clear, several studies indicate that a fall in individuals’ serotonin increases instances of aggression.^15^ Moreover, those with low serotonin cannot be deterred from aggression as effectively as others can by, say, the threat of punishment because lower serotonin levels make individuals more likely to value harming others in retaliation more than they value their own well-being.^16^ More importantly for the purposes of this discussion, there is evidence that increasing serotonin by the use of SSRIs causes a correlative fall in aggression, particularly in individuals with such tendencies and backgrounds.^17^ When prescribed to offenders, pharmacological agents that increase serotonin levels are therefore expected to fortify their moral powers, enabling them to refrain from reoffending.

Apart from pharmacological interventions, there are a number of neurointerventions referred to as ‘electromagnetic brain stimulation’ (EBS), which promise to have similar effects on individuals’ moral powers; these include deep brain stimulation (DBS), transcranial magnetic stimulation (TMS) and transcranial direct-current stimulation (tDCS).^18^ These interventions are typically used to treat essential tremor and Parkinson’s disease, dystonia, obsessive–compulsive disorder, migraine, and treatment-resistant depression. By targeting specific brain regions, these neurointerventions can affect individuals’ moral cognition, aggression, and impulsivity.^19^ Thus, EBS neurointerventions can alter their capacity to understand the requirements of justice and morality, as well as their motivational and emotional capacity to comply with these requirements.

The fortificationist case for neurointerventions is straightforward. Recall that on my account of the Fortificationist Theory there are three reasons that justify any mode of punishment: the state’s duty to ensure the protection of individuals’ rights and liberties, the state’s duty to promote the social conditions for stability, and the state’s duty to satisfy individuals’ interest in developing their moral powers up to the relevant threshold. These reasons clearly provide *prima facie* support for neurointerventions. If there are cases in which the only or best way for a state to discharge these duties is by administering non-consensual neurointerventions, such as the ones described above, then it seems that this mode of punishment is justified. For example, there may be cases in which neurointerventions that increase one’s empathy offer the only, or most effective, or most cost-effective way of fortifying one’s moral powers.^20^

Of course, the claim that neurointerventions are sometimes permissible as a form of punishment should not be taken to imply that offending is caused purely by biological traits. Rather, it seems likely that criminogenic circumstances, such as neglect, abuse, and growing up in disadvantaged socioeconomic circumstances can affect the neurological characteristics of some persons. In any case, whatever the causal relationship between social and biological factors may be, the above discussion indicates that neurointerventions can often have the desired effect, whether that is an improvement in understanding the requirements of justice or overcoming the *motivational* hurdles that might prevent someone from complying with them.

## III Objections

### A Basic Rights and Liberties

However, it may seem that the *prima facie* justification of neurointerventions is not sufficient to establish an all-things-considered permissibility because they violate basic rights and liberties. Thus, the objection goes, even if we have certain reasons in favour of neurointerventions, these reasons are insufficient and in fact outweighed by our rightly held commitments to protecting key liberties. This is because neurointerventions seem to violate a key liberal commitment, which requires the equal protection of each person’s rights and liberties. If these liberties include freedom of thought and liberty of the person, then it may seem that non-consensual neurointerventions can only be imposed at the cost of violating one’s basic rights and liberties.^21^

However, other modes of punishment, such as incarceration, involve a restriction of offenders’ basic liberties, such as freedom of movement, and yet we do not view this restriction as a violation of offenders’ basic rights. When we refer to liberties, we appeal to the normative sense of freedom, which is, in a sense, conditional. That is, our liberties depend on our having certain rightful claims on them. For instance, although having the freedom to kill others would increase our freedom in a descriptive sense, we do not speak of the liberty to kill others. This is because we have a duty to refrain from killing them, which places limits on our freedom. Consider, for instance, the following example, which I borrow from Victor Tadros:

*Hit Man:* I hire a hit man to kill you, a complete innocent. The only way for you to avert the threat is to pull me in front of you, using me as a shield.^22^

In this scenario, it is permissible for you to use me as a shield because I have the duty to respond to the wrongful harm that I created. Given that I have a duty that restricts the liberties that I would have in the absence of this duty, I cannot object that discharging this duty violates my liberties.^23^

This seems to be the argument that explains the widely-held intuition that incarceration does not violate violent offenders’ liberties. For if my incarceration is justified on the basis of my duty to be punished in this way, it follows that my incarceration does not *violate* my liberty.^24^ Similarly, if I have a duty to undergo neurointerventions, this duty places certain limits to my freedom, which implies that discharging this duty does not *violate* my liberty.^25^ For I do not have the liberty to refuse to satisfy my duty to fortify my moral powers.

The duty to undergo neurointerventions springs from the offender’s duty to fortify her moral powers. This duty is enforceable for the three reasons that were appealed to in the previous section. First, states have a duty to protect their citizens’ rights and liberties; when a citizen violates others’ rights, the state ought to take on that citizen’s fortificational duties to ensure the protection of citizens’ rights. Second, all states and citizens have an interest in promoting their society’s stability, which is often undermined by some citizens’ failure to fortify their moral powers. Third, all citizens have an interest in developing their own moral powers. These three reasons are collectively sufficient to establish that citizens’ fortificational duties are enforceable. And, as I have argued, these duties sometimes give rise to the duty to undergo neurointerventions.

### B Proportionality

One might object that even if the above argument can justify restricting some liberties by incurring the cost of incarceration, it is not sufficient to justify non-consensual neurointerventions because this type of punishment can never be proportionate to the aims that it serves. For instance, it would not be permissible to impose a life sentence on someone who has the desire to steal chewing gum because this sentence is disproportionate to the aim of fortifying one’s moral powers against stealing chewing gum.^26^ Similarly, it might be argued that neurointerventions are disproportionate to the aims of punishment as moral fortification.

The proportionality objection in this context can take a number of forms. First, one worry might be that, like most medical treatments, some neurointerventions tend to have harmful side-effects. For example, CPA and MPA anti-libidinal pharmacological agents can cause ‘osteoporosis, weight gain, male breast enlargement, and hot flushes, deep venous blood clots, and subsequent complications’.^27^ Of course, any kind of punishment must be proportionate to the aims that justify it. This account of the proportionality worry implies that the bar of proportionality is set higher with regard to neurointerventions that cause significant side-effects than it is with those that have minor or no side-effects. Moreover, when both options are available and equally effective, the latter should be preferred over the former. For instance, whenever anti-libidinal pharmacological agents are appropriate, GnRH antagonists and SSRIs are to be preferred over CPA and MPA agents, given that the former cause significantly fewer and less significant side-effects.^28^ Thus, the proportionality objection so understood fails to establish that neurointerventions are always disproportionate but it does remind us that the side-effects that a punishment might have ought to be taken into consideration when assessing whether it is proportionate to fortificationist aims.

A different reading of the proportionality objection states that interfering with one’s mental freedom in the way that neurointerventions do infringes one’s autonomy to an extent that renders them disproportionate to the aim of fortifying offenders’ moral powers. One reply to this is to dispute that neurointerventions necessarily infringe individuals’ autonomy: because most offenders offend due to violent impulses or desires they cannot control, neurointerventions will often increase their autonomy by giving them the capacity to act in accordance with their considered judgments.^29^ Yet even if we grant that, in some cases, neurointerventions restrict one’s autonomy, this may be proportionate to the aims of punishment. For, in some cases, the *prima facie* wrongness of infringing autonomy is not sufficient to imply all-things-considered wrongness. Notice, for instance, that even though using me as a shield in *Hit Man* infringes my autonomy, we think that it is permissible for you to use me in this way because the harm that you impose on me is proportionate to the aim of using me (i.e. to avoid the deadly threat that I imposed on you). To see why neurointerventions would be similarly permissible, consider the following variation of *Hit Man*:

*Hit Man injection*: I hire a hit man to kill you, a complete innocent. The only way for you to avert the threat is to pull a lever that will inject me with an empathy-inducing drug, which will make me want to stop the hit man.

The fact that it would be permissible for you to inject me with a neurointervention implies that there are at least some cases in which infringing someone’s autonomy by interfering with their mental freedom is proportionate to the aims of punishment as moral fortification. Given that we would not think that you are permitted to inject me if I had hired someone to steal your chewing gum, we can conclude that the autonomy infringing aspect of neurointerventions should be taken into consideration when assessing their proportionality. Thus, neurointerventions should only be used in cases where very serious crimes have been committed, and only if less autonomy-restricting interventions, such as education and counselling, are not likely to fortify one’s moral powers to the extent that or as quickly as it is required.^30^

Lastly, a related variation of the proportionality objection is that when neurointerventions change who we are so much that there is no psychological continuity between our past and future selves, they are effectively a type of death sentence. Even if we grant that undergoing a neurointervention is equivalent to dying, it seems to follow that if you are permitted to use me as a shield in *Hit Man* in virtue of my duties, then you are also permitted to ask me to undergo neurointerventions in *Hit Man injection*, even if the neurointervention will affect my empathy to such an extent that I will no longer be the same person. We can dispute, however, the claim that neurointerventions are equivalent to death. First, all neurointerventions described in Sect. II require constant treatment and are, therefore, reversible. Moreover, neurointerventions may add or alter a characteristic, emotion or motivation, yet it is far from clear that they cause a complete disruption in one’s psychological continuity. For instance, when similar additions or changes occur due to education, therapy, or medication for depression or anxiety disorders we find it implausible to say that the person that has changed in these ways has died. Therefore, although the proportionality objection reminds us of the significance of ensuring that punishment is always proportionate to the aims that justify it, it does not show that neurointerventions are always disproportionate to all crimes that might be committed.

### C Fortification vs Indoctrination

At this point, it might be argued that if the Fortificationist Theory of Punishment implies that it is permissible for a state to impose non-consensual neurointerventions, then it also, implausibly, implies that nonconsensual indoctrination is permissible, since this could also increase one’s moral powers.

Howard seeks to resist the permissibility of indoctrination by arguing that we need to distinguish between ‘approaches that empower offenders as moral agents – that fortify their moral powers – and those that bypass them’ (Howard 2017, 66). Drawing on the example of Alex in Anthony Burgess’s *A Clockwork Orange*, he suggests that

the problem with the conditioning Alex receives is not that it deprives him of his freedom of what to think – he retains the reflective capacity to affirm convictions – but that it fails to attend to the actual root of the problem: Alex’s attitudes toward his fellow human beings. He is moved to refrain from violating others’ rights simply because he is averse to feeling ill – not because he has grasped, and effectively been moved by, an appreciation of others’ value.^31^

It might seem that, to avoid rendering the Fortificationist Theory implausible, we will need to accept Howard’s response here. But it might seem that this commits us also to rejecting non-consensual neurointerventions.

However, there are two distinct reasons why this response fails to show that neurointerventions are impermissible. First, when Howard states that indoctrination fails to address ‘Alex’s attitude toward his fellow human beings’ he implies that indoctrination bypasses one’s moral powers in the sense that it seeks to produce certain actions without regard to the motivations that people have for performing those actions. This is not the case with certain neurointerventions, however, which only target precisely a person’s attitudes towards others. Second, indoctrination may seek to inculcate a *particular* motivation in someone in order to achieve the right result. For example, Alex’s conditioning has ensured that his motivation for acting in the required way is his aversion to feeling ill. Yet this does not seem to be the case with some neurointerventions, which enable an individual to have access to the *set* of reasonable motivations. The following example makes these differences clearer:

Suppose there is proof of David’s failure to discharge his fortificational duties because we find out that he has assaulted transgender people. David is found guilty of the crimes and sentenced to incarceration. Upon his release from prison, there are good reasons to believe that David’s views have not changed at all but that he would only commit transphobic crimes in the future if he truly thought that he wouldn’t get caught. If David is indoctrinated at this point, he will not commit any hate crimes in the future, but he will not identify with his decision not to act unjustly. If asked whether he believes that hate crimes are wrong, he will not be able to justify his (negative) answer in any way that reasonable persons should accept. By contrast, if David is subjected to deep brain stimulation, his empathy could be increased. As a result, he would be able to place himself in other people’s shoes and consider things from their point of view, without having his judgment clouded by extreme, irrational emotions, such as hatred. In this case, if asked whether he believes that hate crimes are wrong, David would be able to give appropriate reasons in defence of his (negative) answer.

Now one might respond that both interventions are impermissible because David should fortify his moral powers on his own, or by being given reasons that he can consider and evaluate. Bublitz and Merkel, for example, seem to be making the second claim when they argue that, by acting directly on the brain, ‘direct interventions seem to violate the demands of dignity’ and that indirect interventions, on the other hand, such as ‘speech or sounds or images, engage with the other’s first-person perspective by recognizing and referring to her beliefs and feelings’.^32^ The thought here is that dignity requires treating persons as agents by respecting their ‘first-person perspective’, which neurointerventions and any other kind of direct intervention cannot achieve. This claim, however, is quite controversial, given that certain forms of brainwashing that may involve forcing someone to watch or listen to something, for instance, are *indirect* interventions, yet arguably violate the demands of dignity at least as much as direct interventions do. Similarly, there are cases of *direct* interventions, such as certain kinds of psychological rehabilitation that may involve psychiatric treatment, which seem permissible; this intuition is even stronger when we compare these interventions to *indirect* kinds of brainwashing. Thus, we may conclude that respecting the ‘first-person perspective’ is neither sufficient nor necessary for the permissibility of an intervention, and that the distinction between direct and indirect interventions only tracks our intuitions regarding the moral and physical boundaries that bodies seem to set, and not the extent to which an intervention shows lack of respect towards human agency. Instead, I have suggested that a more plausible criterion is whether an intervention targets one’s moral powers as a capacity rather than specific beliefs or desires.

To be sure, neurointerventions might not be sufficient as a means for increasing one’s moral powers to the requisite degree. For instance, many sex offenders do not offend because they have a high sex drive but because they have certain desires that involve harming others and certain views about, for example, women’s worth. In these cases, even if chemical castration prevents further crimes, it might not enhance the offenders’ moral powers, as they are likely to have the same desires and views post-castration. Of course, as I argued in the previous section, this is not the case with all offenders. If offending is due to impulses as contrasted with more stable desires, then giving an individual the capacity to control these impulses can actually increase that person’s autonomy and enable them to act in accordance with their true desires. A good way to distinguish between the two categories of offenders might be by appealing to the difference between an offender’s first-order and second-order desires.^33^ In cases where the first-order desire to offend is different from the offender’s *reasonable* second-order desire, neurointerventions might be sufficient to bring the two into alignment. By contrast, when both the offender’s first-order and second-order desires are inconsistent with the requirements of justice, it seems likely that extensive therapy and education will be necessary. In any case, the fortificationist argument in favour of neurointerventions is compatible with the claim that neurointerventions are not always sufficient means to ensure the required fortification; in fact, it offers reasons in favour of other methods of rehabilitation as well, such as therapy and education.

## IV Conclusion

I have argued that the fortification of criminal offenders’ moral powers through non-consensual neurointerventions is justifiable for three reasons. First, when a citizen violates others’ rights due to her failure to discharge her fortificational duties, the state is permitted to enforce those duties on the basis of its duty to protect individuals’ rights and liberties. One way to do this is by requiring that individual to undergo neurointerventions. Second, the state’s enforcement of fortificational duties is required for the assurance of others’ compliance with the requirements of justice, which underpins societal stability. In these cases, neurointerventions may be warranted if they are the only or best way of promoting citizens’ stable and fair social cooperation. Third, because all persons have an interest in developing their moral powers to the degree required in order to participate in social cooperation, the state has a duty to ensure that this moral power can be developed. This interest establishes a third reason in favour of neurointerventions.

In certain cases, these reasons may be outweighed by competing considerations, such as considerations of effectiveness, cost, and the dangers associated with increasing the power that unjust states possess in our non-ideal world. However, even though the reasons that we have in favour of neurointerventions are by no means conclusive in a non-ideal framework, they do suggest that such uses of criminal justice interventions are consistent with the Fortificationist Theory of Punishment and that there may be cases in which such interventions are not only morally permissible but required.

## Footnotes

1 See, for example, William M. Burdon and Catherine A. Gallagher (2002) ‘Coercion and Sex Offenders: Controlling Sex-Offending Behavior Through Incapacitation and Treatment’, *Criminal Justice and Behaviour*, 29 (1), 87–109; Focquaert, Farah (2014) ‘Mandatory Neurotechnological Treatment: Ethical Issues’, *Theoretical Medicine and Bioethics*, 35 (1), 59–72. See Sect. II for a more elaborate discussion of different neurointerventions.

2 See Jeffrey Howard (2017) ‘Punishment as Moral Fortification’, *Law and Philosophy*, 36 (1), 45–75.

3 Howard, ‘Punishment as Moral Fortification’, p. 45.

4 Howard, ‘Punishment as Moral Fortification’, p. 48.

5 Howard, ‘Punishment as Moral Fortification’, p. 45.

6 For the distinction between epistemic and motivational reasonableness, see Howard, ‘Punishment as Moral Fortification’, p. 49. For a similar distinction between philosophical and political reasonableness, see Erin Kelly and Lionel McPherson (2001) ‘On Tolerating the Unreasonable’, *The Journal of Political Philosophy*, 9 (1), 38–55, at pp. 39–40.

7 For an anti-perfectionist argument of this kind, see John Rawls (2005) *Political Liberalism: Expanded Edition*. New York: Columbia University Press, p. 77.

8 See, for example, Jonathan Quong (2011) *Liberalism without Perfection*. Oxford: Oxford University Press, p. 305: ‘if we assume a central purpose of liberal justice is to provide a fair framework within which citizens can develop and exercise their moral powers’, then an unreasonable kind of education is not consistent with this purpose and so ‘there may be good grounds for intervention’. For the latter claim, see Sophia Isako Wong (2010) ‘Duties of Justice to Citizens with Cognitive Disabilities’, in E. F. Kittay and L. Carlson (eds.), *Cognitive Disability and its Challenge to Moral Philosophy*. Oxford: Wiley-Blackwell.

9 Howard, ‘Punishment as Moral Fortification’, p. 54.

10 See Harvey Gordon and Don Grubin (2004) ‘Psychiatric Aspects of the Assessment and Treatment of Sex Offenders’, *Advances in Psychiatric Treatment*, 10 (1), 73–80; Christopher Chew, Thomas Douglas, and Nadira Faber (2018) ‘Biological Interventions for Crime Prevention’, in D. Birks and T. Douglas (eds.) *Treatment for Crime: Philosophical Essays on Neurointerventions in Criminal Justice*. Oxford: Oxford University Press.

11 Deepak Prabhakar and Richard Balon (2010) ‘How Do SSRIs Cause Sexual Dysfunction?’, *Current Psychiatry*, 9 (12), 30–34.

12 For example, the Oregon Depo-Provera trial studied the effects of MPA agents on three groups of sexual offenders. The first consisted of offenders who underwent treatment, the second had offenders who were prescribed MPA but did not undergo treatment, and the third contained offenders who were not eligible for prescription. The trial indicated that only 5% of those in the first group re-offended, and none of the offences were sexual, while 26–30% of the other two groups re-offended, and half of the offences were sexual. Barry M. Maletzky and Gary Field (2003) ‘The Biological Treatment of Dangerous Sexual Offenders: A Review and Preliminary Report of the Oregon Pilot Depo-Provera Program’, *Aggression and Violent Behavior*, 8 (4), 391–412.

13 Chew et al., ‘Biological Interventions for Crime Prevention’.

14 Chew et al., ‘Biological Interventions for Crime Prevention’.

15 Donald M. Dougherty, James M. Bjork, Dawn M. Marsh, and F. Gerard Moeller (1999) ‘Influence of Trait Hostility on Tryptophan Depletion-Induced Laboratory Aggression’, *Psychiatry Research*, 88 (3), 227–232; F. Gerard Moeller, Donald M. Dougherty, Alan C. Swann, Diana Collins, Chester M. Davis, and Don R. Cherek (1996) ‘Tryptophan Depletion and Aggressive Responding in Healthy Males’, *Psychopharmacology*, 126 (2), 97–103; A. J. Cleare and A. J. Bond (1995) ‘The Effect of Tryptophan Depletion and Enhancement on Subjective and Behavioural Aggression in Normal Male Subjects’, *Psychopharmacology*, 118 (1), 72–81.

16 Molly J. Crockett, Annemieke Apergis-Schoute, Benedikt Herrmann, Matthew D. Lieberman, Ulrich Müller, Trevor W. Robbins, and Luke Clark (2013) ‘Serotonin Modulates Striatal Responses to Fairness and Retaliation in Humans’, *The Journal of Neuroscience*, 33 (8), 3505–3513.

17 Chew et al., ‘Biological Interventions for Crime Prevention’.

18 These techniques involve applying ‘magnetic fields or electrical currents to specific areas of the brain, with the aim of modulating brain activity. DBS involves the invasive neurosurgical placement of metal electrodes within the brain to transmit electrical impulses. TMS and tDCS, on the other hand, non-invasively induce changes in brain activity through the use of superficial magnets and electrodes respectively. These two modes have a shallow depth of effective penetration relative to DBS and are thus only able to affect relatively superficial brain regions Recent developments in TMS technology, though, suggest it may be possible to extend this range’. Chew et al., ‘Biological Interventions for Crime Prevention’.

19 Chew et al., ‘Biological Interventions for Crime Prevention’.

20 It should be stressed that, according to this view, variation between different individuals’ moral powers does not matter when it occurs above the threshold that is required for one’s participation in social cooperation. It follows that even if non-consensual neurointerventions are permissible in order to raise the moral powers of someone who has not fortified sufficiently, it is not permissible to use them to fortify the moral powers of someone who has already discharged her fortificational duties. For more on the issue of the problem of variation, see Richard Arneson (1999) ‘What, if Anything, Renders All Human Beings Morally Equal?’, in Dale Jamieson (ed.), *Singer and His Critics*. Oxford: Blackwell; Ian Carter (2011) ‘Respect and the Basis of Equality’, *Ethics*, 121 (3), 538–571; Ian Carter (2013) ‘Basic Equality and the Site of Egalitarian Justice’, *Economics & Philosophy*, 29 (1), 21–41; and Christopher Nathan (2011) ‘Need There Be a Defence of Equality?’, *Res Publica*, 17, 211–225.

21 See Christoph Bublitz (2016) ‘Moral Enhancement and Mental Freedom’, *Journal of Applied Philosophy*, 33 (1), 88–106.

22 Victor Tadros (2011) *The Ends of Harm: The Moral Foundations of Criminal Law*. Oxford: Oxford University Press, p. 267.

23 I am grateful to an anonymous reviewer for this point.

24 Tadros justifies this duty in deterrent terms: on his view, if I harm someone wrongfully, I have the duty to bear the cost of punishment in order to prevent others from harming others in similar ways. See Tadros, *The Ends of Harm*, p. 277. This duty is cast in very different terms in the Fortificationist Theory, as it is linked to an offender’s fortificational duties. Thus, justifying incarceration in the context of punishment as fortification is considerably more difficult, given that the aim is not deterrence but, rather, the fortification of offenders’ moral powers.

25 Notice that accepting this argument does not require us to accept that we have a duty to be punished by incarceration, as the previous sentence states, precisely because incarceration typically fails to enable offenders to fortify their moral powers.

26 I thank an anonymous reviewer for pressing the proportionality objection and for raising this example.

27 Chew et al., ‘Biological Interventions for Crime Prevention’; Frederico D. Garcia and Florence Thibaut (2011) ‘Current Concepts in the Pharmacotherapy of Paraphilias’, *Drugs*, 71 (6), 771–790; Peer Briken and Martin P. Kafka (2007) ‘Pharmacological Treatments for Paraphilic Patients and Sexual Offenders’, *Current Opinion in Psychiatry*, 20 (6), 609–613.

28 Chew et al., ‘Biological Interventions for Crime Prevention’.

29 Thomas Søbirk Petersen and Kristian Kragh (2017) ‘Should Violent Offenders Be Forced to Undergo Neurotechnological Treatment? A Critical Discussion of the ‘Freedom of Thought’ Objection’, *Journal of Medical Ethics*, 43 (1), 30–34.

30 For instance, if you could make me want to stop the hit man by sharing certain thoughts and reasons with me, then you should prefer that option. Similarly, if you could avert the threat without using me as a shield in *Hit Man*, then you ought to do so.

31 Howard, ‘Punishment as Moral Fortification, p. 66.

32 Jan Christoph Bublitz and Reinhard Merkel (2014) ‘Crimes Against Minds: On Mental Manipulations, Harms and a Human Right to Mental Self-Determination’, *Criminal Law and Philosophy*, 8, 51–77, at p. 73.

33 Harry G. Frankfurt (1971) ‘Freedom of the Will and the Concept of a Person’, *Journal of Philosophy*, 68 (1), 5–20, at p. 7.

